# Classification of EEG Using Adaptive SVM Classifier with CSP and Online Recursive Independent Component Analysis

**DOI:** 10.3390/s22197596

**Published:** 2022-10-07

**Authors:** Mary Judith Antony, Baghavathi Priya Sankaralingam, Rakesh Kumar Mahendran, Akber Abid Gardezi, Muhammad Shafiq, Jin-Ghoo Choi, Habib Hamam

**Affiliations:** 1 Department of Computer Science and Engineering, Loyola-ICAM College of Engineering and Technology, Chennai 600034, India; 2 Department of Computer Science and Engineering, Rajalakshmi Engineering College, Chennai 602105, India; 3 Department of Electronics and Communication Engineering, Veltech Multitech Dr. Rangarajan Dr. Sakunthala Engineering College, Chennai 600062, India; 4 Department of Computer Science, COMSATS University Islamabad, Islamabad 45550, Pakistan; 5 Department of Information and Communication Engineering, Yeungnam University, Gyeongsan 38541, Korea; 6 Faculty of Engineering, Uni de Moncton, Moncton, NB E1A 3E9, Canada; 7 International Institute of Technology and Management, Commune d’Akanda, BP, Libreville 1989, Gabon; 8 School of Electrical and Electronic Engineering Science, Department of Electrical Engineering, University of Johannesburg, Johannesburg 2006, South Africa; 9 Spectrum of Knowledge Production & Skills Development, Sfax 3027, Tunisia

**Keywords:** electroencephalogram, adaptive classifier, support vector machine, common spatial pattern, online recursive independent component analysis

## Abstract

An efficient feature extraction method for two classes of electroencephalography (EEG) is demonstrated using Common Spatial Patterns (CSP) with optimal spatial filters. However, the effects of artifacts and non-stationary uncertainty are more pronounced when CSP filtering is used. Furthermore, traditional CSP methods lack frequency domain information and require many input channels. Therefore, to overcome this shortcoming, a feature extraction method based on Online Recursive Independent Component Analysis (ORICA)-CSP is proposed. For EEG-based brain—computer interfaces (BCIs), especially online and real-time BCIs, the most widely used classifiers used to be linear discriminant analysis (LDA) and support vector machines (SVM). Previous evaluations clearly show that SVMs generally outperform other classifiers in terms of performance. In this case, Adaptive Support Vector Machine (A-SVM) is used for classification together with the ORICA-CSP method. The results are promising, and the experiments are performed on EEG data of 4 classes’ motor images, namely Dataset 2a of BCI Competition IV.

## 1. Introduction

Several EEG signal feature extraction methods have been introduced in recent years. These feature extraction methods include Empirical Mode Decomposition, Autoregressive approaches, Wavelet transform methods, Phase-Space Reconstruction Approach, and CSP-based methods [1]. In this article [2] combined the methods of Variational Mode Decomposition (VMD) and Hilbert Transform (HT) to extract valuable characteristics from EEG recordings and the stacking neural network to identify epilepsy seizures to suggest an intelligent system. The HT approach extracts characteristics from EEG signals after the VMD method decomposes EEG signals into intrinsic mode functions. Using extracted features, the stacked-NN method is used to identify epilepsy seizures [3]. A Common Spatial Pattern (CSP), one of the well-liked and effective approaches for Motor Imagery (MI) Brain Computer Interface (BCI), was used to extract features for the categorization of motor-imaging tasks [4,5]. This approach provides spatial filters that enable separation of two conditions by optimizing variance differences between them. CSP filters are perfectly suited to identify mental states that are characterized by motor sensory rhythm effects since the variance of band-pass filtered signals is equivalent to band-power [6].

Despite being highly efficient, the CSP approach is noise sensitive for small datasets [7]. In attempt to address this problem, a number of CSP variations have been proposed that boost its robustness. In [8] this proposed work the Common Spatio-Spectral Pattern (CSSP) to enhance the performance of CSP even more. Within CSP in CSSP, a finite impulse response filter has been optimized in this approach. This is accomplished by including a temporal delay, which improves CSSP performance and makes it possible to specify separate frequency filters. The CSSSP (Common Sparse Spectral Spatial Pattern) methodology was suggested to improve the CSSP method [9].This strategy finds spectral patterns that are common to all the channels rather than finding unique spectral patterns for each channel as in CSSP.

The technique known as the Sub-Band Common Spatial Pattern (SBCSP), in which the motor imagery EEG signals are filtered at different sub-bands and CSP features are extracted from each of the sub-bands [10] and in this work [11], created the Filter Bank CSP (FBCSP) to undertake autonomous selection of significant temporal-spatial discriminative EEG properties in order to address this problem. SBCSP, however, has not taken into account the probable correlation of the CSP characteristics collected from multiple sub-bands. Then, CSP characteristics are retrieved from each of the bands created by bandpass-filtering the EEG readings into different ranges of frequency. Automatically choosing discriminative pairings of frequency bands and matching CSP features is then done using a feature selection method. FBCSP performed better than SBCSP, but it also used more sub-bands, which raised the computational cost.

The authors proposed utilizing the Discriminant Filter Bank CSP (DFBCSP) to choose the highest discriminant sub-bands from a number of overlapping sub-bands [12]. DFBCSP improved classification accuracy while using less CPU power than SBCSP and FBCSP. The spatial patterns produced by the CSP algorithm draw attention to the underlying neural activity that is crucial for differentiating between different motor tasks. The phase discrepancies between spatial places are not explicitly treated by CSP, which is a drawback. According to [13,14], the phase can provide important information for identifying the different forms of motor imagery activity.

The Analytical Common Spatial Pattern (ACSP), which enables the definition of magnitude and phase features, was used for the first time [15]. By choosing a group of spatial filters that maximise variance for one class of data while reducing variance for the other, the ACSP approach aims to discriminate between two classes of data. As opposed to its real-valued cousin, ACSP can handle complex-valued variance, which may be more illuminating. To achieve the best results, the authors [16] suggested using a manually calibrated decimation filter. The dimensionality of the features was decreased using Fishers’ Discriminant Analysis (FDA), and an SVM classifier was deployed. The technique (known as CD-CSP-FDA) produced improved performance compared to cutting-edge alternatives. The multiple filter band Sparse Filter Bank CSP (SFBCSP) method, Ref. [17] introduced, is used to optimize the sparse patterns. Significant CSP characteristics are chosen from many overlapping frequency bands using a supervised approach. Then, using the chosen characteristics, an SVM classifier is utilized to categorize motor images.

Sparse Bayesian learning, which has been applied for feature selection in a variety of applications, has also lately attracted more interest. The decomposition of the EEG signal into several sub-bands and extraction of CSP characteristics [18]. The Bayesian learning technique is utilized to create sparse features, and the SVM classifier is then employed for classification. Empirical averaging of covariance matrices for training samples is carried out in CSP. This includes the poor signal quality, which reduces system performance. As a result. In [19], presented a sparsity-aware technique that added weighted averaging. Weight coefficients are allocated to each of the trial. The efficiency of the CSP algorithm was enhanced by using this weighting approach to calculate the average covariance matrix.

As the CSP approach needs a lot of electrodes to obtain good results, According to [20], introduced a unique feature extraction technique called common spatial patterns with autoregressive parameters to boost the CSP classification accuracy with less electrodes (CSP-AR). The CSP-AR approach optimizes the differences between two populations in addition to making explicit use of frequency data (i.e., right and left motor imagery). The test results reveal that the CSP-AR has a higher classification accuracy of 87.1 percent than standard CSP and AR parameters, which is demonstrated using the motor imagery data set from the second BCI Competition. The CSP-AR technique improves classification results while simultaneously offering the advantages of high robustness.

A linear classifier establishes classification boundaries based on the value of a linear combination of variables or features. Linear Discriminant Analysis (LDA) and Support Vector Machines (SVMs) are the most often used classifiers in EEG-based BCIs, especially in online and real-time BCIs [21,22]. SVM often outperforms competing classifiers [23].

The classification methods used in EEG-based BCIs may be broken down into four categories: transfer learning, deep learning, adaptive classifiers, grid, and tensor classifiers. It is clear that, even for unsupervised adaptation, adaptive classifiers outperform static ones. By modifying the classifier online, fresh data entered while using the BCI may be used for ongoing training of the classifier, minimizing the amount of training data needed while also enhancing execution by enhancing the classifier’s ability to alter data. When fresh EEG data become available for adaptive classifiers, the settings are continually reviewed and changed. As a result, it is suggested to pair the ORICA-CSP based feature extraction approach with an Adaptive Support Vector Machine classifier. The suggested technique is used to differentiate between four motor imagery tasks using actual EEG data from nine human individuals.

The remaining paper is organized as follows: Section 2 explains the Adaptive SVM classifier and the framework of A-SVM with ORICA is explained in Section 3. The experimental results of the proposed approach are discussed in Section 4 and finally the work is concluded in Section 5.

## 2. Adaptive SVM Classifier

The standard SVM is a non-probabilistic binary linear classifier, for example prediction is done for every information that is given and identifies where between the two classes is the information [24]. An assumption is made by SVM that the information sources are numeric. In the event where categorical information sources are present, they must be changed to binary dummy variables (one variable for every classification). SVM can do linear classification and work as a nonlinear classification using kernel tricks where the inputs are mapped to high-dimensional feature spaces. In taking care of the nonlinear classification problem of SVM, the kernel function is utilized rather than the internal product calculation and nonlinear problems are changed over to linear classification problems by increasing their dimensionality.

Consider a training set of the form ($m_{1},\;n_{1}$)……($m_{n}$,$n_{n}$), where $m_{i}$ belongs to the class $n_{i}$ that is represented as 1 or −1. SVM uses a hyperplane to segregate the datasets $m_{i}$ with $n_{i}$ as 1 from the datasets $m_{i}$ with $n_{i}$ being −1. Datasets $\;m_{i}$ are expanded; it is said to be the maximum margin hyperplane; this is done when the distance of the hyperplane is closest. The hyperplane is written as v. m−b=0, where v is a normal vector. The offset of the hyperplane along the vector can be determined through the parameter $\;\frac{b}{\left||v|\right|}$. To maximize the distances between the planes, ||v|| needs to be maximized.

Adaptive classifiers with progressively updated online parameters were developed to address EEG non-stationarity and monitor changes in EEG features over time. Additionally, by learning online, adaptive classifiers can function with little to no offline training data. Due to the non-stationarity of brain signals, the adaptive processing can lessen accuracy loss in the subsequent classification step as well as modest changes in the global mean during the course of evaluation sessions. Since these oscillations have nothing to do with the activity at hand, they can be handled without supervision. The same classification model may be used for training and assessment sessions.

Guided user training is necessary for supervised BCI adaptation, during which the users’ orders are enforced and the appropriate EEG class labels are therefore known. With free BCI use, supervised adaptation is not feasible since the real label of the incoming EEG data is unknown. The entering EEG data’s label is unknown with unsupervised adaptation. As a result, unsupervised adaptation is based on an estimation of the data class labels for retraining or updating, or it is based on class-unspecific adaptation, such as updating the classifier model with the general all classes EEG data mean.

The weights given to each feature in a linear discriminant hyperplane, for example, are adaptive classifier parameters that are progressively re-estimated and modified over time when fresh EEG data become available. As a result, even with non-stationary inputs like an EEG, the classifier can follow potentially changing feature distribution and continue to function well. Both supervised and unsupervised adaptation, or knowing the actual class labels of the incoming data, may be used by adaptive classifiers. In supervised adaptation, the real class labels of the entering EEG signals are known, and the classifier is either updated solely using the new data and retrained using the existing training data enriched with the new, labelled incoming data.

## 3. A-SVM with ORICA-CSP Framework

It is proposed to pair the ORICA-CSP based feature extraction approach with an adaptive support vector machine classifier. The suggested technique is used to differentiate between four motor imagery tasks using actual EEG data from nine human individuals. In an MI movement, the applicant imagines how the actual action would be executed, and the EEG modality records the appropriate neuro-electric processes. There are several varieties of EEG-based BCI, including single finger motions from one hand utilizing human EEG signals, continuous arm movement using EEG signals, simple and compound limb motor imagery, etc. The majority of research studies in the current MI literature are concerned with two-class or three-class problems, such as left hand vs. right hand and left hand, right hand, and feet, respectively. In this case, the two feet are treated as a single class. When two-foot movement needs to be discriminated, between multiclass MI movement or more than three class classification work, is always a difficult issue.

### 3.1. System Architecture

**Figure 1:**
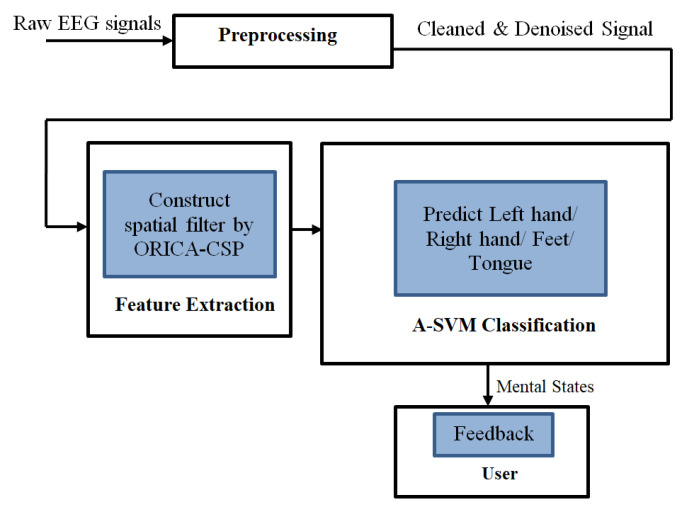
System architecture.

The overall system architecture is represented in Figure 1. The figure describes the workflow with the proposed feature extraction and classification approaches. Initially, the raw EEG signals are preprocessed with the ORICA approach to remove the signal artifacts and the Common Spatial Pattern filter is generated to extract the features, which is then sent for classification by the proposed Adaptive SVM based approach that classifies the signals into four classes as Left hand, Right hand, Feet, and Tongue. This approach of signal processing is explained further in detail.

### 3.2. Dataset Description

The BCI COMP IV 2a dataset, which was created from data collected from nine people in the BNCI Horizon 2020 database, served as the basis for the Motor Imagery EEG data used in this study. Four motor imagining tasks were included in the cue-based BCI paradigm: left hand (class 1), right hand (class 2), both feet (class 3), and tongue movement (class 4). Two separate sessions were videotaped for each subject on several days. In each session, six runs were spaced apart by brief pauses. At the beginning of each session, a recording of around five minutes was produced to gauge the EOG impact. Three segments of the tape were used: two minutes of open eyes (gazing at a fixation cross on the screen), one minute of closed eyes, and one minute of eye movements.

Twenty-two Ag/AgCl electrodes with 3.5 cm between them were used to capture the EEG. The left mastoid served as the reference and the right mastoid as the ground while all signals were collected monopolarly. Data between 0.5 and 100 Hz were bandpass filtered after being recorded at 250 Hz. The sensitivity of the amplifier was set at 100 volts. An extra 50 Hz notch filter was turned on to lessen line noise. Along with the 22 EEG channels, three monopolar EOG channels were also captured and sampled at 250 Hz. They had a 0.5 to 100 Hz bandpass filter applied to them (with the 50 Hz notch filter enabled), and the amplifier’s sensitivity was set at 1 mV [25].

### 3.3. ORICA

To enable the ORICA algorithm and CSP appropriate for MI EEG data feature extraction, a novel framework is developed. The original sources may be successfully retrieved using this framework and supplied to CSP as input for feature extraction. The ICA algorithm’s goal is to identify the de-mixing matrix M^−1^, where M is the mixing matrix in the recorded EEG signal, and then to recover the EEG sources. The independent source signals may combine into dependent signals during the mixing process. The whitening procedure is thought to address this issue by making the source independent by lowering the correlation between the signals. The whitening process and the separation process, where the whitened signals are applied for the de-mixing process, are therefore two processes that make up the separation process.

The recordings will be separated into blocks, with the same number of samples in each block, in order to simplify processing and make the method more universal and usable. Instead of repeating the iteration for every sample, it will be done in brief blocks of samples. The number of block sizes affects how quickly the method converges. The effective duration of the time frame is determined by the forgetting factor. The appropriate window length will be short if the value of the forgetting factor is high. A large beginning value of the forgetting factor is often required to achieve rapid convergence, whereas a lower starting value is employed to reduce variation.

To make the mixed sources independent, the signals after pre-processing are subjected to the online whitening process. The artifact-contaminated MI EEG recording source is accurately and successfully separated using the ORICA algorithm [26]. For each iteration, the whitening matrix *X* and the de-mixing matrix *W* are computed according to Equations (1) and (3), respectively, by adding the block-update rule on matrix *W*. Since the iteration is processed in blocks instead of each sample, the computational complexity can be reduced:

(1) $$X_{i+1}=X_{i}+\frac{u_{i}}{1-u_{i}}\;\left[I-\frac{v_{i}\times v_{i}^{T}}{1+u_{i}\left(v_{i}^{T}\times v_{i}-1\right)}\right]\;\;\times X_{i}$$

where *X_i_*, Whitening matrix; *i*, number of iterations; $v_{i}=X_{i}x_{i}$, the whitened data; $u_{i}$, factor to be forgotten; *I*, an identity matrix:

(2) $$W_{i+1}=W_{i}+l_{r}\;\left[I-f\left(a_{i}\right)\times a_{i}^{T}\right]\times W_{i}$$

(3) $$W_{i+1}=W_{i}+\frac{u_{i}}{1-u_{i}}\;\left[I-\frac{a_{i}\times f^{T}\left(a_{i}\right)}{1+u_{i}\left(f^{T}(a_{i}-1\right)}\right]\;\times \;W_{i}$$

where $W=M^{-1}$, demixing matrix $a{\;}_{i}=W_{i}v_{i}$; $\;l_{r}$, learning rate; $f\left(a_{i}\right)$, activation function.

Original EEG and EOG sources are restored once ORICA is applied. When people do MI, just one specific area of the brain is activated. The contralateral areas over the motor cortex, for instance, are active during hand imaging. The mid-central or parietal regions are engaged in images of the feet and tongue. However, it is unknown which of the separated components corresponds to the active region of the brain.

### 3.4. ORICA-CSP

Consider using the ORICA-processed characteristic elements of a motor imaging process in an experiment as the input for the CSP algorithm. Calculations should be made using the CSP feature extraction approach, and all feature vectors should be chosen to create a spatial filter *W* for multiclass EEG data. Apply the aforementioned filter *W* on data1 and use the filtered results as data 2. Do the math to get the data energy of the number of independent components—also known as the number of channels.

To create a new filter *W*′, the feature vector that best represents the energy difference between the various categories in each training set is chosen. Feature extraction is done when filtering is done using the enhanced filter *W*′. The final features for classification are acquired after performing a logarithmic transformation on the feature values due to the significant variance between particular feature values. The ORICA-CSP working is explained in the Algorithm 1.
**Algorithm 1:** ORICA-CSP algorithm.**Input** decomposed EEG signal using HOL-SSA **for** I = 1 to n **Compute**
$X_{i+1}=X_{i}+\frac{u_{i}}{1-u_{I}}\;\left[I-\frac{v_{i}*v_{i}^{T}}{1+u_{i}\left(v_{i}^{T}{*v}_{i}-1\right)}\right]$* I // whitening matrix **Compute**
${\;W}_{i+1}=W_{i}+\frac{u_{i}}{1-I_{i}}\;\left[I-\frac{a_{i}{*f}^{T}\left(a_{i}\right)}{1+u_{i}\left(f^{T}(a_{i}-1\right)}\right]$* I // demixing matrix **end** **for** k=1 to 4 **Compute** $S_{k}={\;U}_{K}U_{K}^{T}$ / trace ($U_{K}U_{K}^{T}$) $U_{o}\;\Delta U_{o}^{T}$// diagonal decomposition M =$\Delta^{-1/2}$ $U_{o}^{T}$// whitening matrix **Compute**
$W_{k}$ and $W_{k}^{\prime }$**//** whitening covariance matrices $S_{k}$ and $S_{k}^{\prime }$ $W_{k}={\;\mathrm{MS}}_{k}M^{T}$ $W_{k}^{\prime }={\;\mathrm{MS}}_{k}^{\prime }M^{T}$ **Compute**
$P_{k}={\;U}_{k}^{T}\;*\;M$ **Decompose** $S_{k}$= $D_{k}/P_{\mathrm{ks}}$ **Generate**
$P_{s}=\overset{4}{\underset{k=1}{\sum }}P_{\mathrm{ks}}$ **end** **for** trial t=1 to n **Compute**
$f_{v}=\log(V_{m}/\underset{t=1}{\sum }V_{m})$// $V_{m},$ variance matrix **end**

The separated sources are sent for feature extraction through the construction of a spatial filter *W* for multiclass EEG data, using the filter *W* to select the most obvious feature vector of the energy difference between different categories in each training set to form a new filter *W*’. This is done after the whitening matrix *X* and the de-mixing matrix *W* have been computed in accordance with Equations (2) and (3), respectively. The final features for classification are acquired after performing a logarithmic transformation on the feature values because of the significant variance between particular feature values.

Current brain–computer interfaces raise concerns about the non-stationarity of the underlying signals. It becomes difficult to transfer a classifier from one session to the next as a result, and the need for input sample collection at the conclusion of each session results. Employing an adaptive classifier is one way to maintain performance while lowering the likelihood of the training required for ideal BCI performance. It is suggested to use an adaptive classification method based on support vector machines.

Due to its effective classification performance, versatility in handling multi-dimensional data, and explicit error control, SVM is a popular classification paradigm in BCI systems. The fundamental goal of SVM is to build hyperplanes with the highest possible classification accuracy by reducing the cost function and maximizing the margins between classes. Support vectors display hyperplanes.

The main advantage of SVM is that it can be used as both an inconsistent and a consistent classifier. SVM may be turned into an inconsistent classifier by using one of the numerous kinds of kernel functions available, such as polynomial, radial basis, and sigmoid functions. In the current investigation, a sigmoid function was employed. Platt’s probabilistic output is used to determine the sigmoid function that calculates the posterior class probabilities $v_{j}$. Since it has been shown to outperform consistent classifiers in terms of classification accuracy, an inconsistent SVM was chosen. The accuracy of the BCI system may be significantly higher after adaptation than it would be without adaptation, showing that online BCI adaptation improves performance. The Algorithm 2 below explains how the adaptive SVM works with the ORICA-CSP feature extraction approach.
**Algorithm 2:** A-SVM with ORICA-CSP.**Step 1: Input** features vectors
$f_{v}=\log(V_{m}/\underset{t=1}{\sum }V_{m})$ $//V_{m}$, variance matrix of EEG signal projection **Step 2: Determine** the class label with function $f(s_{i}$$)=(w,\;s_{i}$) + b $//s_{i}$ ∈ Rn, with N samples {I$,\ldots ,\;s_{N}$ } **Step 3: Classify **
$\mathrm{the}\;\mathrm{new}\;\mathrm{sample}\;\mathrm{by}\;u_{j}$ $\;\mathrm{and}\;v_{j}$ //$u_{j}$$=\mathrm{sign}(f(s_{i}$$))\;\mathrm{and}\;\mathrm{the}\;\mathrm{posterior}\;\mathrm{class}\;\mathrm{probability},\;v_{j}$$=\mathrm{Pb}(v_{j}$$=v_{j}$$|s_{i}$) is calculated using Platt’s probabilistic output. $//\mathrm{Classifier}\;\mathrm{is}\;\mathrm{adapted}\;\mathrm{with}\;u_{j}$ $\mathrm{and}\;v_{j}$ **Step 4: Define** threshold th **Step 5:**$\;\mathrm{if}\;v_{j}$ $>\;\mathrm{th}\;\mathrm{holIs},\;s_{i}$ is introduced to the dataset for training T **Step 6: Update** whenever new samples are included in the solution

To avoid completely retraining each iteration, we adopted an incremental training strategy. Every time a new sample is added, the adaptive SVM will progressively update the solution. Due to its increased speed and ability to handle high-dimensional data, the adaptive classifier may now be employed in an online setting. The algorithm above explains how the Adaptive SVM works in terms of classification. Determine the class labels using the training data samples $s_{i}$ as indicated in step 2. If the posterior class probability is higher than the threshold set, the classifier should be updated with the new data when new samples like $u_{j}$ and $v_{j}$ are discovered.

## 4. Results and Discussion

The signal variable comprises 25 channels that are processed between 8 and 12 Hz (the latter three are EOG signals, while the first 22 are EEG signals). The EOG channels should not be utilized for classification; instead, they should be used for artifact processing operations thereafter. Here, the artifact removal strategy is the ORICA method.

The accuracy of the classifier and the associated Information Transfer Rate (ITR) were determined in order to assess the results from several sessions and for the various adaption approaches.

Although the ITR is a commonly used indicator of BCI performance that takes into account a better correlation of different BCI frameworks, it is a way for evaluating the BCI execution that ignores the actual plan of the BCI application and will thus often overestimate the performance. ITR and accuracy are used to gauge how well the suggested technique is working.

**Figure 2:**
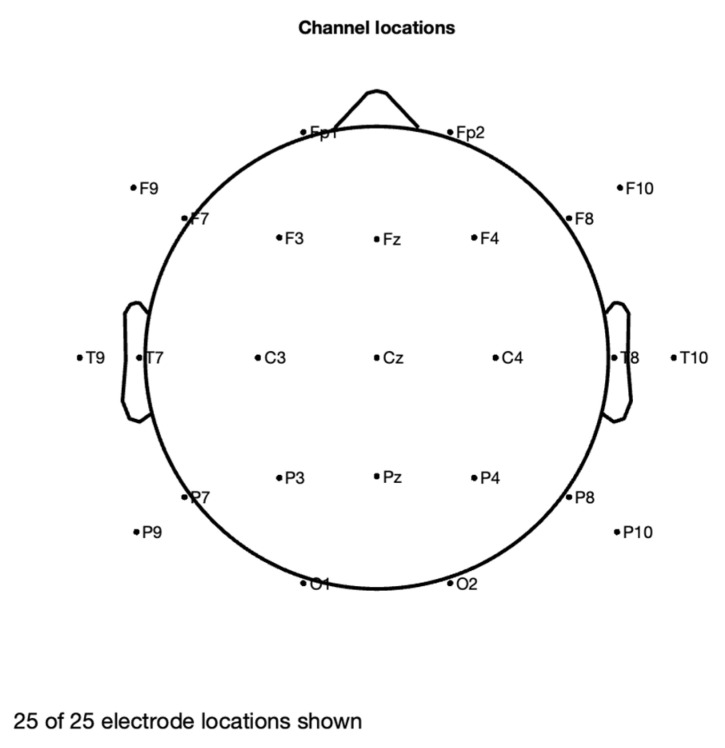
Channel locations.

**Figure 3:**
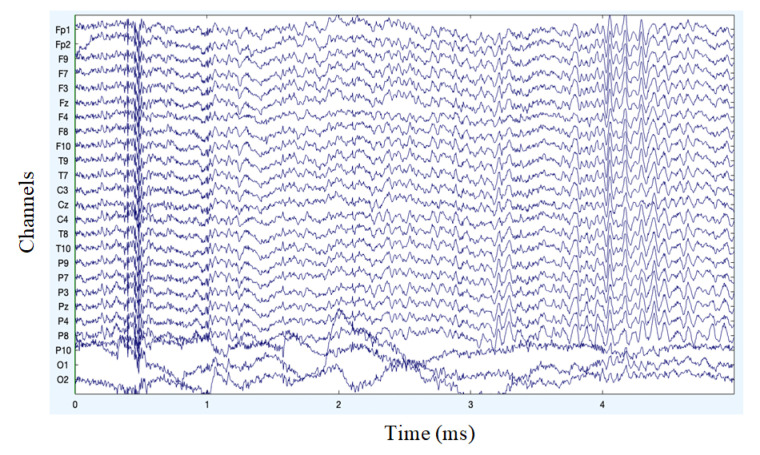
Original channel data of MI signals.

**Figure 4:**
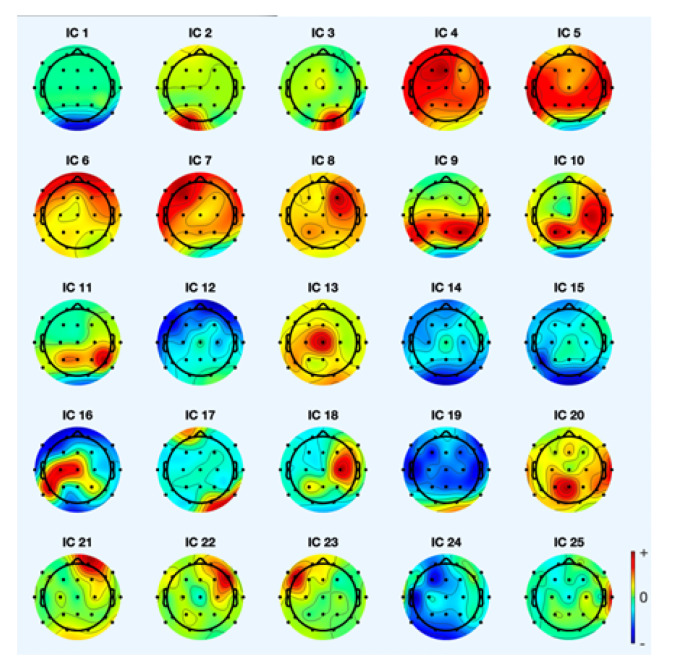
Topoplots of the independent components.

The analysis of artifact removal from the motor imagery signals using the proposed method is explained below. Figure 2 represents the channels used to acquire the motor imagery signals and their locations. The signals acquired by each of these channels are represented in Figure 3 as channel data. These signals are further decomposed using the proposed decomposition approach and the topoplots of all the independent components are shown in Figure 4.

**Figure 5:**
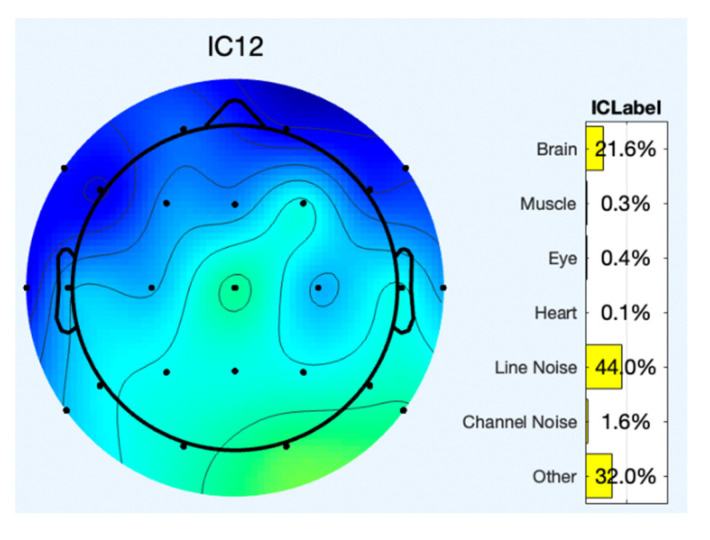
Component 12 with brain signals and other signals.

**Figure 6:**
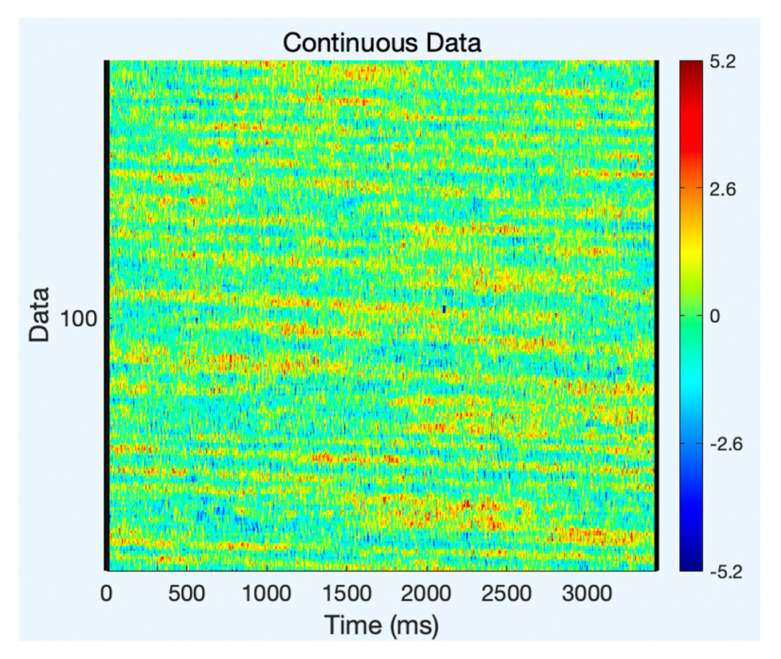
ERP of component 12.

A sample of two component’s topoplot is explained below for analysis. Figure 5 depicts the topoplot of component 12 and the presence of brain signals and other artifacts. The ERP (Event Related Potential) of component 12 is represented in Figure 6. Similarly, the presence of EEG signals and artifacts in the other components are shown in Table 1.

**Figure 7:**
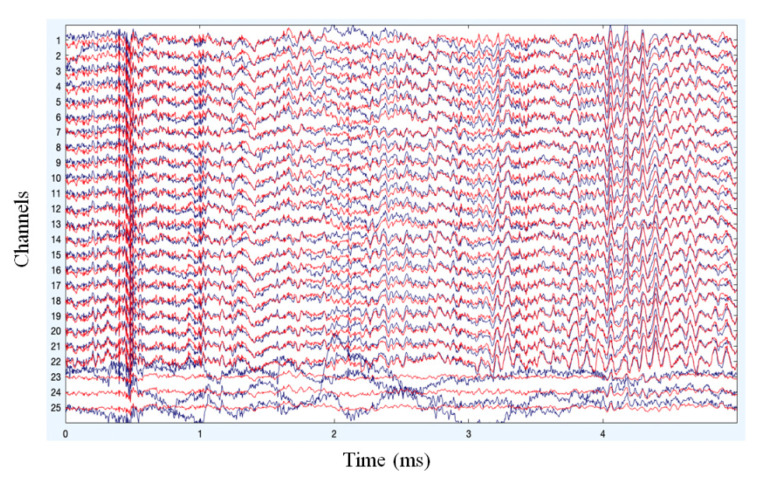
Pruned data after artifact removal.

With the Online Recursive Independent Component Analysis approach of artifact removal, the original source signal is separated from the other artifact signals. This separation of signal is represented in Figure 7 where the signals in red denote the artifact free EEG signals.

The categorization of imaginary motor tasks for EEG-based BCI using the ORICA-CSP feature extraction method is the focus of this work. The effectiveness of the suggested method is evaluated using dataset 2a from the BCI Competition IV’s multiclass problem. The performances were contrasted with those of the other publicly available approaches, CSP and ICA + Wavelet-CSP. According to our study, using the ORICA+CSP feature extraction approach in combination with adaptive classification would boost the classification accuracy since it provided much higher kappa values than other methods, as will be detailed below.

**Figure 8:**
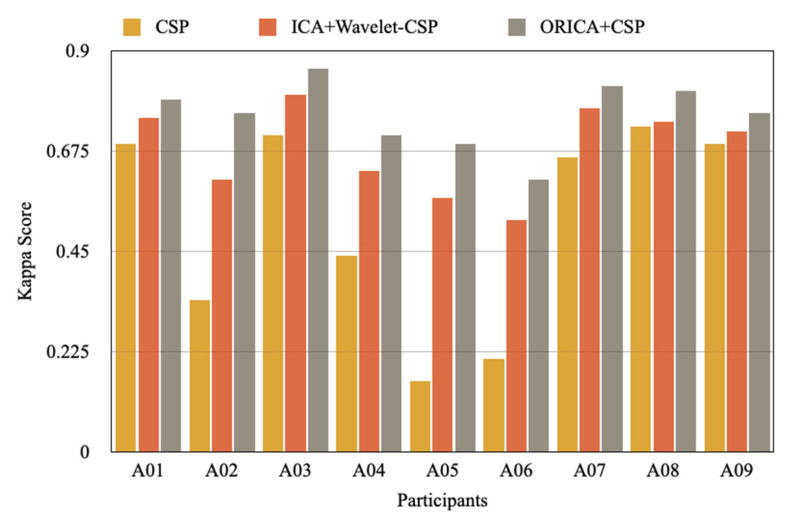
Performance comparison in terms of Cohen’s Kappa coefficient.

**Figure 9:**
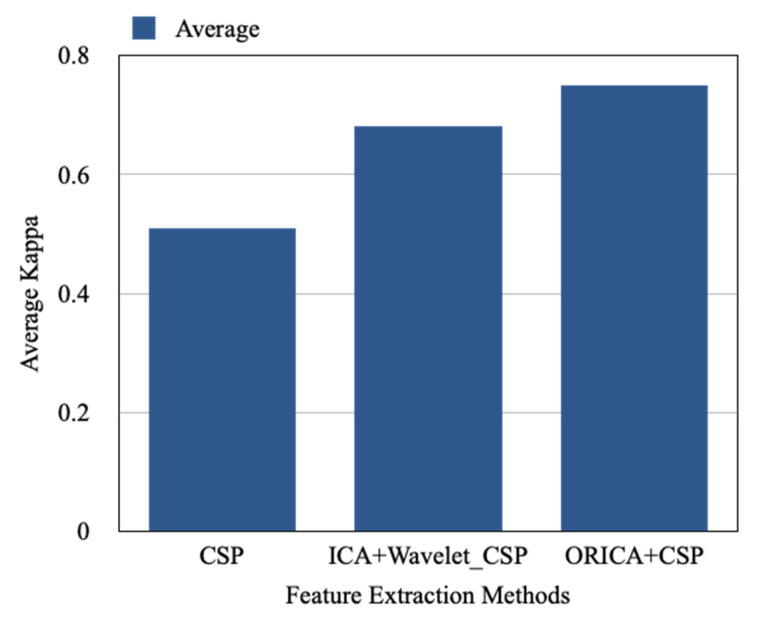
Comparison of average kappa value with CSP, ICA + Wavelet-CSP, and ORICA + CSP.

The results of the suggested approach are compared to those of the Wavelet-CSP on the BCI Competition IV 2a and the results of the standard CSP based on the 8–30 Hz IIR band-pass filters (4 class motor imagery dataset). A kappa coefficient (k) was utilized in this competition as a criterion of uniqueness. Table 2 displays the k values for the two techniques as well as the suggested strategy. In comparison to Wavelet-CSP and traditional CSP with a band-pass filter, the suggested technique yields an average k value of 0.75 rather than 0.68 and 0.51, respectively. Figure 8 also shows the performance analysis of each participant using a different feature extraction approach, and Figure 9 compares the average results.

The results of the proposed method are compared to those of the ORICA-CSP method, which produces better results because it computes complex-valued spatial filters instead of choosing a wavelet function that necessitates the extraction of sources’ prior knowledge, as opposed to the Wavelet-CSP method. This enhancement raises the possibility that spatial filters derived from ORICA-CSP might offer more information on the interactions between different cortical areas when mental activities are being carried out. Our findings essentially indicate that ORICA-CSP produced a more reliable motor imaging feature extraction than the conventional CSP and Wavelet-CSP methods. This indicates that a greater categorization success rate may result from the suggested strategy. The major cause of this situation is the noise sensitivity of traditional CSP.

The signal quality change will be accompanied by variations in the classification success rate. Studies have shown a frequency overlap between the artifacts and the motor imagery signals. Since the Wavelet-CSP depends on the chosen wavelet function and an 8–30 Hz IIR band-pass filter cannot only eliminate certain distortions but also runs the risk of damaging motor imagery signals, choosing a wavelet function necessitates extracting previous knowledge from the sources.

However, by isolating the motor imagery signals from the raw EEG to eliminate the artifacts, the ICA approach may better preserve the integral of the motor-related signals. However, the ORICA method outperforms the conventional ICA in terms of efficiency when compared to high-density EEG data. This suggests that the ORICA algorithm is a viable technique for live time series identification since it has the capacity to adapt to the immediate mixing. Therefore, the suggested approaches may retain accurate classification results even when the raw EEG is of low quality.

The results in Table 3 are the comparative analysis of the LDA, SVM, Adaptive LDA, and Adaptive SVM based classification approaches applied on the acquired motor imagery signals from nine subjects. In addition, the corresponding Information Transfer Rate (ITR) for the accuracy achieved is represented in Table 4.

**Figure 10:**
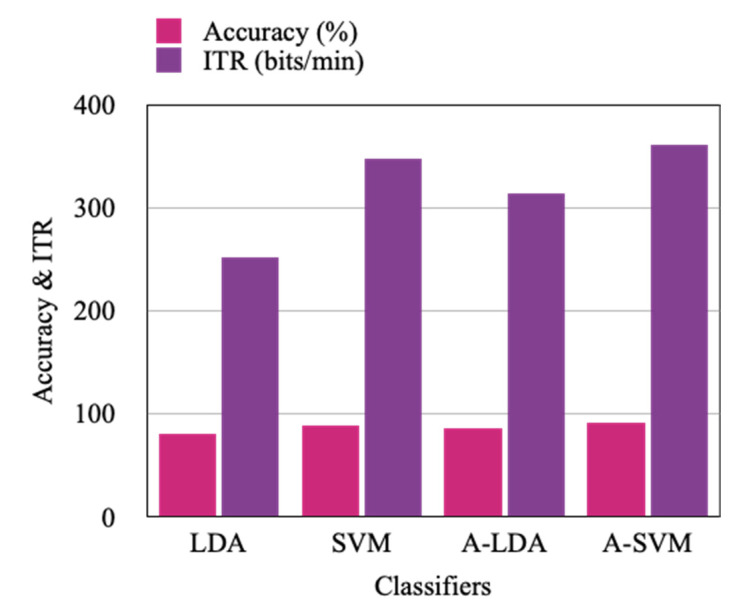
Subjects’ average classification comparison in terms of accuracy and ITR.

This analysis is also represented in Figure 10. From this experimentation, it is observed that Adaptive SVM based classification achieves better accuracy with an average of 91% compared to the linear SVM, LDA, and Adaptive LDA classification which resulted in an accuracy of 89%, 81%, and 86%, respectively, with the proposed approach of ORICA-CSP based feature extraction where ORICA-CSP seems to perform better with all the classifiers used for analysis. Similarly, the Information Transfer Rate represented in Table 4 reports Adaptive SVM with the highest rate of 360.38 bits/min, whereas the other classifiers used in the comparative study linear SVM, LDA, and Adaptive LDA have achieved 347.10 bits/min, 251.80 bits/min, and 313.38 bits/min, respectively.

**Figure 11:**
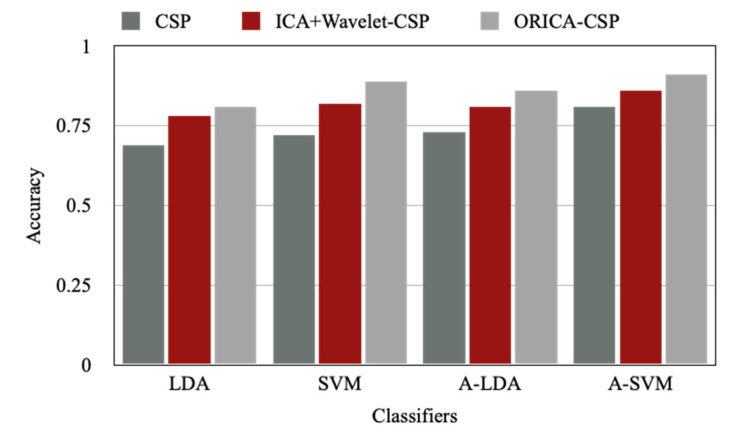
Classifier comparison chart with different feature extraction methods.

The classification of the same motor imagery signals achieved with the LDA, SVM, A-LDA, and A-SVM with different existing and proposed feature extraction methods of CSP, ICA+Wavelet-CSP, and ORICA-CSP are presented in Table 5. It is observed from the results that the Adaptive Support Vector Machine and other classifiers achieve the highest accuracy with the proposed ORICA-CSP approach of feature extraction compared to the CSP and ICA+Wavelet-CSP approach. This comparison analysis is explained graphically in Figure 11.

**Figure 12:**
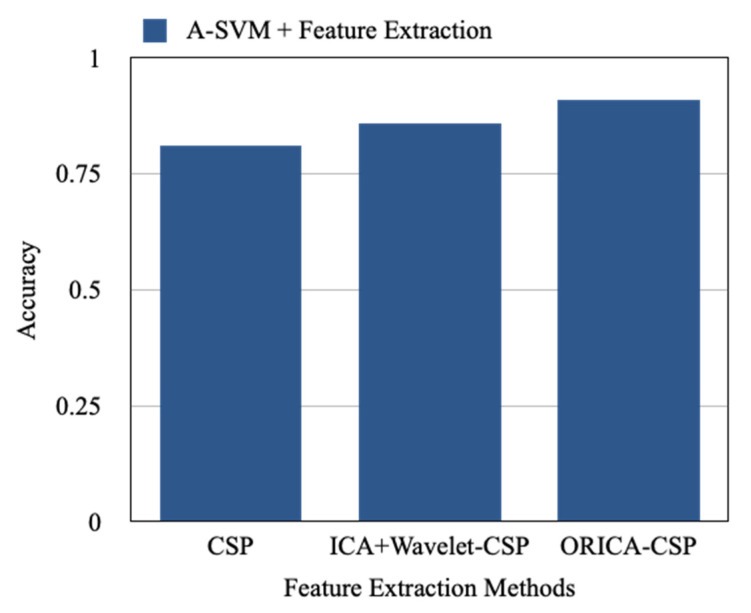
Average accuracy of A-SVM with various feature extraction methods.

**Figure 13:**
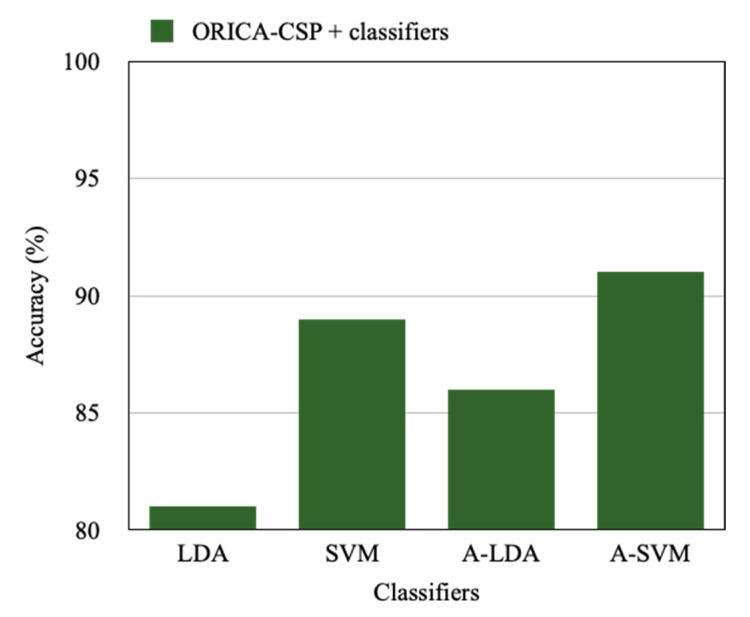
Comparative results of the proposed feature extraction method with different classifiers.

Highlighting the proposed results, Figure 12 is constructed to represent the accuracy achieved by the Adaptive Support Vector Machine with the CSP, ICA-Wavelet-CSP, and ORICA-CSP feature extraction approaches which are 0.81, 0.86, and 0.91, respectively, where A-SVM seems to perform better with ORICA-CSP comparatively. Similarly, Figure 13 depicts the ORICA-CSP feature extraction approach applied on the LDA, SVM, A-LDA, and A-SVM methods, which resulted in an accuracy of 0.81, 0.89, 0.86, and 0.91, respectively. It is observed that ORICA-CSP with LDA performs much better comparatively.

## 5. Conclusions

The proposed work attempted to use the adaptive classifier on the Motor Imagery based BCI which was not tried earlier in the past. The classifier used was an adaptive SVM classifier implemented on the BCI COMP IV 2a, four class MI EEG signals. The benefits of the ORICA-CSP feature extraction method are combined with the Adaptive SVM based classifier in the proposed work to optimize the classification accuracy and the information transfer rate. The Adaptive SVM classifier proved to produce better accuracy compared to the existing LDA and adaptive LDA based classifiers as LDAs are not highly applicable on nonlinear problems and as they are preferred only for a smaller number of samples. Similarly, the accuracy of the ORICA-CSP feature extraction method is compared with other classifiers to check the accuracy level that has been achieved for the four class MI signals. The experimentation is carried with different combinations of the discussed feature extraction and classification techniques. Upon conclusion, it is observed that the proposed Adaptive SVM with the ORICA-CSP feature extraction method is found to result in an improved accuracy and rate of information transfer on the preferred MI. The work is planned to be enhanced under two phase classification in future.

## Figures and Tables

**Table 1:** EEG and artifacts present in the observed signals.

Components/Signals	EEG (%)	Muscle (%)	Eye (%)	Heart (%)	Line Noise (%)	Channel Noise (%)	Other (%)
**IC 1**	37.1	7.0	0.2	1.0	3.9	2.5	48.2
**IC 2**	6.9	26.5	0.6	0.1	2.1	2.7	61.1
**IC 3**	4.9	38.7	0.0	0.2	1.5	0.7	53.9
**IC 4**	91.7	0.3	0.0	0.1	4.4	0.0	3.6
**IC 5**	39.8	0.4	0.1	0.8	16.2	0.0	42.7
**IC 6**	78.1	1.5	0.2	1.3	0.5	0.7	17.6
**IC 7**	73.6	1.4	0.0	0.3	12.2	0.1	12.5
**IC 8**	42	0.8	0.2	0.2	2.5	0.5	21.5
**IC 9**	91.7	0.1	0.0	0.1	4.1	0.0	3.9
**IC 10**	67.0	0.0	0.0	0.0	8.3	0.0	24.7
**IC 11**	91.0	0.0	0.0	0.1	0.9	0.0	8.0
**IC 12**	21.6	0.3	0.4	0.1	44.0	1.6	32.0
**IC 13**	84.3	8.5	0.1	0.4	1.7	0.1	4.9
**IC 14**	43.4	0.5	0.1	3.2	36.5	3.4	12.9
**IC 15**	6.1	0.9	0.1	3.1	3.9	5.3	80.4
**IC 16**	99.1	0.0	0.0	0.6	0.1	0.0	0.2
**IC 17**	12.3	3.2	2.6	0.3	5.9	0.7	75.0
**IC 18**	95.8	0.0	0.0	0.0	2.2	0.1	1.9
**IC 19**	0.5	0.1	0.0	1.4	5.5	0.2	92.2
**IC 20**	42.5	0.8	0.1	0.2	16.6	0.1	39.8
**IC 21**	2.5	0.9	9.6	0.2	8.3	0.8	77.8
**IC 22**	5.2	2.0	8.9	0.2	2.7	5.1	75.9
**IC 23**	5.2	2.0	8.8	0.2	2.7	5.1	76.1
**IC 24**	1.6	0.3	0.3	0.5	31.2	0.5	65.7
**IC 25**	4.5	3.2	0.2	6.0	5.9	0.6	79.6

**Table 2:** Performance comparison of every participant in terms of Cohen’s Kappa coefficient.

Subject/Participants	CSP	ICA + Wavelet-CSP	ORICA + CSP
A01	0.69	0.75	0.79
A02	0.34	0.61	0.76
A03	0.71	0.80	0.86
A04	0.44	0.63	0.71
A05	0.16	0.57	0.69
A06	0.21	0.52	0.61
A07	0.66	0.77	0.82
A08	0.73	0.74	0.81
A09	0.69	0.72	0.76
Mean	0.51	0.68	0.75

**Table 3:** Results of LDA, SVM, A-LDA, and A-SVM in terms of accuracy on Motor Imagery data.

Subjects/Participants	LDA (%)	SVM (%)	A-LDA (%)	A-SVM (%)
A01	79	90	85.6	90.1
A02	79.5	88.8	89.3	91.3
A03	81.1	87.6	87	88
A04	81.7	87.2	82.2	90.8
A05	75.9	88.9	84.8	92.2
A06	79.8	87.8	85.8	85.9
A07	80.9	89.2	85.7	89.2
A08	79.8	86.5	86.9	88.9
A09	78.2	90.5	82.6	89.6
Mean	81	89	86	91

**Table 4:** Results of LDA, SVM, A-LDA, and A-SVM in terms of ITR (bits/min) on Motor Imagery data.

Subject/Participants	LDA	SVM	A-LDA	A-SVM
A01	246.39	365.33	313.33	366.60
A02	251.08	350.44	356.57	382.16
A03	266.46	336.11	329.13	340.83
A04	272.39	331.44	277.41	375.60
A05	218.62	351.66	304.58	394.29
A06	253.91	338.46	315.55	316.66
A07	264.50	355.34	314.44	355.34
A08	253.91	323.42	327.98	351.66
A09	239.02	371.71	281.47	360.30
Mean	251.808	347.101	313.384	360.382

**Table 5:** The average classification accuracy of several feature extraction techniques is compared.

Feature Extraction/Classifier	LDA	SVM	A-LDA	A-SVM
**CSP**	0.69	0.72	0.73	0.81
**ICA-Wavelet-CSP**	0.78	0.82	0.81	0.86
**ORICA-CSP**	0.81	0.89	0.86	0.91

## Data Availability

Not applicable.
